# Microbial extraction of chitin from seafood waste using sugars derived from fruit waste-stream

**DOI:** 10.1186/s13568-020-0954-7

**Published:** 2020-01-28

**Authors:** Yun Nian Tan, Pei Pei Lee, Wei Ning Chen

**Affiliations:** School of Chemical and Biomedical Engineering, Nanyang Technological University, 62 Nanyang Drive, Singapore, 637459 Singapore

**Keywords:** Chitin, Chitosan, Prawn shell, Lactobacillus, Fermentation, Carbon source

## Abstract

Chitin and chitosan are natural amino polysaccharides that have exceptional biocompatibility in a wide range of applications such as drug delivery carriers, antibacterial agents and food stabilizers. However, conventional chemical extraction methods of chitin from marine waste are costly and hazardous to the environment. Here we report a study where shrimp waste was co-fermented with *Lactobacillus plantarum* subsp. *plantarum ATCC 14917* and *Bacillus subtilis* subsp. *subtilis ATCC 6051* and chitin was successfully extracted after deproteinization and demineralization of the prawn shells. The glucose supplementation for fermentation was replaced by waste substrates to reduce cost and maximize waste utilization. A total of 10 carbon sources were explored, namely sugarcane molasses, light corn syrup, red grape pomace, white grape pomace, apple peel, pineapple peel and core, potato peel, mango peel, banana peel and sweet potato peel. The extracted chitin was chemically characterized by Fourier Transform Infrared Spectroscopy (FTIR) to measure the degree of acetylation, elemental analysis (EA) to measure the carbon/nitrogen ratio and X-ray diffraction (XRD) to measure the degree of crystallinity. A comparison of the quality of the crude extracted chitin was made between the different waste substrates used for fermentation and the experimental results showed that the waste substrates generally make a suitable replacement for glucose in the fermentation process. Red grape pomace resulted in recovery of chitin with a degree of deacetylation of 72.90%, a carbon/nitrogen ratio of 6.85 and a degree of crystallinity of 95.54%. These achieved values were found to be comparable with and even surpassed commercial chitin.

## Introduction

The rising affluence of Asia in recent years has ushered an insatiable demand for seafood consumption and heralded a boom in the seafood processing industry worldwide (Yadav et al. 2019). With the development of improvised and sophisticated techniques in aquaculture, shellfish production such as shrimp, crab and krill have increased exponentially (Hamed et al. 2016). As such, 6–8 million tons of crustacean waste are generated annually, as 45–60% of shrimp comprising of head, shell and tail portions are discarded as processing by-products (Mao et al. 2017). The huge increase in shrimp waste has sparked an interest in the extraction of chitin which is the most abundant polysaccharide in the marine ecosystem (Tarafdar and Biswas 2013). The chemical composition of shrimp waste is estimated to be 20–30% chitin, 20–40% protein, 30–60% minerals (calcium carbonate) and 0–14% lipids (muscle residues and carotenoids) (Kaur and Dhillon 2015; Shahidi and Synowiecki 1991).

Chitin, the second most abundant biopolymer in nature after cellulose, is a linear poly-β-(1,4)-N-acetyl-d-glucosamine occurring in ordered crystalline microfibrils (Al Sagheer et al. 2009). It appears in 3 polymorphic forms: α-chitin, β-chitin and γ-chitin (Cardenas et al. 2004). In α-chitin, which is the most stable and common configuration, polysaccharide chains are arranged in anti-parallel strands allowing maximum bonds, resulting in chitin fibrils with high crystallinity index of 80% (Sarbon et al. 2015). α-chitin is found in insect cuticles, crustacean shells and the cell walls of yeast and fungus (Sujeeetha et al. 2015). β-Chitin, which occurs in squid pens and extracellular fibers of diatoms, is arranged in parallel chains, resulting in less stability with a 70% crystallinity index of chitin fibrils (Alabaraoye et al. 2018). Due to the larger distance between neighboring polymer chains, β-chitin is more reactive and dissolves readily (Tripathi and Singh 2018). γ-Chitin, which is rare, is a combination of both α-chitin and β-chitin (Hayes et al. 2008).

Chitin extraction from shellfish comprises deproteinization to remove proteins and demineralization to remove the inorganic calcium carbonate on the shell (Khanafari et al. 2008; Wahyuntari and Setyahadi 2011). Shrimps have a thinner shell wall than lobsters and crabs (Thirunavukkarasu and Shanmugam 2009; Bautista et al. 2001), thus extraction efficiency is highest using shrimp waste compared to other crustacean waste (Percot et al. 2003). Chemical and enzymatic treatments exist to prepare pure chitin (Beaney et al. 2004). However, chemical deproteinization using sodium hydroxide (NaOH) results in partial deacetylation of chitin and hydrolysis of the biopolymer, lowering its molecular weight (Younes and Rinaudo 2015). Chemical demineralization using dilute hydrochloric acid (HCl) have also been reported to result in chitin with high residual ash content and polymer degradation (Islam et al. 2011). As high temperatures and high acid concentrations in chemical treatments have frequently resulted in chitin deacetylation and depolymerization (Charoenvuttitham et al. 2006), biological treatments are preferred to preserve the chitin structure with a high degree of deacetylation and molecular weight, while being free of proteins and minerals (Sedaghat et al. 2017; Kim and Park 2015). In addition, smaller solvent input is required in the biological extraction of chitin, resulting in simpler manipulation, lower energy consumption, and higher reproducibility in a shorter turn-around time. This in turn also leads to lesser chemical by-products to be discharged in wastewater effluent (Arbia et al. 2013; Gortari and Hours 2013).

The production of lactic acid from *Lactobacillus plantarum* subsp. *plantarum ATCC 14917* and proteolytic enzymes from *Bacillus subtilis* subsp. *subtilis ATCC 6051* have been found to remove calcium carbonate and protein successfully and extract chitin effectively during shell waste fermentation (Harkin et al. 2015; Hajji et al. 2015). In our study, we have chosen a co-culture fermentation strategy over a two-step successive mono-culture so that the metabolic pathways of all bacteria strains involved can be synergistically harnessed (Liu et al. 2014; Aytekin and Elibol 2010). Due to the development of green extraction techniques as well as the push to decrease the cost of chitin purification, we have decided to explore the use of carbon-rich waste as a substrate to replace the glucose supplement in the fermentation process (Adour et al. 2008; Thakur et al. 2019). This is because production volume and processing costs can be reduced with a high concentration sugar waste substrate as a carbon source (Bayrak and Buyukkileci 2018; Tropea et al. 2014). Simultaneous saccharification and fermentation also avoids the addition of expensive hydrolytic enzymes such as cellulases and amylases which are usually required to break down complex substrates containing cellulose (Sadh et al. 2018; Panesar and Kaur 2015).

To the best of our knowledge, little information has been reported on the structural characteristics of extracted chitin when shell waste fermentation is simultaneously carried out with the utilization of lignocellulose biomass hydrolysates to facilitate microbial production at a more competitive cost (Paul et al. 2014). Hence this study aims to carry out structural characterization with the use of Fourier Transform Infrared Spectroscopy (FTIR) to measure the degree of deacetylation, elemental analysis (EA) to measure the carbon/nitrogen ratio and X-ray diffraction (XRD) to obtain the degree of crystallinity (de Queiroz et al. 2017). Subsequently, the results are analyzed to evaluate if the change in carbon source has a statistically significant impact on the extracted chitin structure.

## Materials and methods

### Fermentation of prawn shell waste

*Lactobacillus plantarum* subsp. *plantarum ATCC 14917* was inoculated in 5 mL of De Man, Rogosa and Sharpe (MRS) broth and incubated overnight at 200 rev min^−1^ and 37 °C, while *Bacillus subtilis* subsp. *subtilis ATCC 6051* was inoculated in 5 mL of Luria broth (LB) and incubated overnight at 200 rev min^−1^ and 30 °C. The following day, 5 g of prawn shell waste was added to a conical flask, which was covered with a cloth cap and autoclaved at 121 °C. 100 mL of 20% sterile glucose solution (1:20 w/v ratio) was then added to the autoclaved prawn waste. The optical densities (OD) of the bacteria strains grown were measured to determine concentration. The desired concentration of culture (1 × 10^6^ colony forming units (CFU) per milli-liter of overnight culture) was then inoculated into the sterile fermentation flask and incubated at 200 rev min^−1^ and 30 °C. After 5 days, the fermented supernatant was filtered off and the prawn shell material washed with deionized water and sterilized with 70% (v/v) ethanol. The prawn shell remains were then dried in a vacuum oven at 60 °C for 24 h before being pound into smaller pieces for analysis.

### Fourier Transform Infrared Spectroscopy (FTIR) analysis

A PerkinElmer Spectrum One Fourier Transform Infrared Spectroscopy (FTIR) was used to characterize the crude chitin samples. The extracted chitin was prepared into a disc by grinding with dried potassium bromide (KBr) and applying a pressure of 10 tons for 2 min. Analysis was performed across the range 4000–500 cm^−1^. The degree of deacetylation (DD%) was calculated using the formula:

$${\text{DD}}\% \, = \,{{\left( {{{{\text{A}}_{ 1 6 50} } \mathord{\left/ {\vphantom {{{\text{A}}_{ 1 6 50} } {{\text{A}}_{ 3 4 50} }}} \right. \kern-0pt} {{\text{A}}_{ 3 4 50} }}} \right)} \mathord{\left/ {\vphantom {{\left( {{{{\text{A}}_{ 1 6 50} } \mathord{\left/ {\vphantom {{{\text{A}}_{ 1 6 50} } {{\text{A}}_{ 3 4 50} }}} \right. \kern-0pt} {{\text{A}}_{ 3 4 50} }}} \right)} { 1. 3 3 { } \times { 1}00}}} \right. \kern-0pt} { 1. 3 3 { } \times { 1}00}}$$

where ‘A’ represents the absorbance of the respective wavenumbers 1650 and 3450 cm^−1^. Amide-I band (1650 cm^−1^) is used as the analytical band and the hydroxyl band (3450 cm^−1^) as the internal reference band. The factor ‘1.33’ denotes the value of the ratio of A_1650_/A_3450_ for fully N-acetylated chitin (Khan et al. 2002). DD% was determined as it reflected the mixture of chitin and chitosan in the extracted crude chitin sample (Sivashankari and Prabaharan 2017). During deacetylation of chitin to chitosan, acetyl groups are removed from the chitin and depolymerization occurs, which is indicated by changes in the molecular weight of chitosan (Qandil et al. 2018).

### Elemental analysis (EA)

An Elementar Vario EL III Elemental Analyzer was used to determine the carbon/nitrogen ratio of the crude chitin extracted. 5 mg of the crude chitin was added to a preformed tin foil boat, after which the boat was folded into a pellet and loaded into a sample carousel. Once dropped into the instrument, the chitin sample undergoes catalytic tube combustion in an oxygenated, high temperature CO_2_ atmosphere. Helium carries carbon and nitrogen through specific adsorption columns where the components are separated and their concentrations determined by a thermal conductivity detector. The carbon/nitrogen ratio was measured to correlate with the decomposition rates of organic material present in the prawn waste by fermentation (Haynes 2014).

### X-ray diffraction (XRD)

X-ray diffraction was performed using a Bruker D2 Phaser. 0.5 g of the crude extracted chitin was placed on a circular sample holder to form a 1 cm diameter before being loaded into the instrument. The voltage and current of the X-ray source were preset to 30 kV and 10 mA and the scan type and scan mode remained unchanged as “Coupled Two Theta/Theta” and “Continuous PSD fast”. The scanning range was set to start at 5° and stop at 80° with a step size increment of 0.1°. The X-rays directed at the crude chitin sample were reflected at an angle θ in accordance to Bragg’s Law and the diffracted waves formed sharp constructive interference peaks into a collected diffraction pattern. The position and intensities of these peaks were then analyzed to identify the underlying structure of the crude extracted chitin. The crystalline index (CrI) was determined by the equation:

$${\text{CrI}}_{ 1 10} \, = \,\left[ {{{\left( {{\text{I}}_{ 1 10} {-}{\text{I}}_{\text{am}} } \right)} \mathord{\left/ {\vphantom {{\left( {{\text{I}}_{ 1 10} {-}{\text{I}}_{\text{am}} } \right)} {{\text{I}}_{ 1 10} }}} \right. \kern-0pt} {{\text{I}}_{ 1 10} }}} \right] \, \times { 1}00$$

where I_110_ is the maximum intensity at 2θ = 20^o^ and I_am_ is the intensity of amorphous diffraction at 2θ = 16^o^ (Liu et al. 2012).

### Change of carbon source

The procedures for fermentation of prawn shell and characterization using the above mentioned spectroscopic methods were repeated but the supplementary 20% glucose solution was replaced with a variety of agro-industrial wastes to serve as the sugar substrate (Brunerová et al. 2017; World Bioenergy Association 2016). A total of 10 carbon sources were explored—namely sugarcane molasses, light corn syrup, red grape pomace, white grape pomace, apple peel, pineapple peel and core, mango peel, banana peel, potato peel, and sweet potato peel. Sugarcane molasses and light corn syrup were purchased off the shelf from the supermarket in the form of pre-made concentrated viscous liquids and diluted with deionized water to make up to a solution of 20% (v/v). Red and white grape pomace were prepared by crushing the grapes to squeeze out the grape juice as done in wineries and collecting the grape skins waste with slight remnants of grape flesh (Dwyer et al. 2014). The pineapple core was removed as performed in pineapple canning factories and mixed together with the discarded pineapple peel before being grinded with a blender into fine pulp (FDA 2019). Apple, mango, banana, potato and sweet potato were relatively straightforward as only peeling of the skins were required. 5 g of prawn waste was added to each of the 100 mL of autoclaved liquid carbon sources, and also to 15 g of the autoclaved solid carbon sources together with 100 mL sterile water and combined with before being inoculated with overnight cultures of *Lactobacillus plantarum* subsp. *plantarum ATCC 14917* and *Bacillus subtilis* subsp. *subtilis ATCC 6051* strains and incubated for 5 days at 200 rev min^−1^ and 30 °C. The extracted crude chitin samples were then subjected to FTIR, EA and XRD analysis.

## Results

### Yield of extracted chitin after fermentation

The dry weights of the extracted crude chitin samples were recorded after fermentation as shown in Table 1. The dry weight of chitin extracted from corn syrup fermentation was the lowest at 0.38 g. Fermentation with apple peel, glucose and banana peel also showed a significant decrease in weight of prawn waste prior fermentation, with dry weights of 0.47 g, 0.50 g and 0.51 g respectively. This was closely followed by fermentation with mango peel and pineapple peel and core with dry weights of 0.55 g and 0.57 g. Chitin extracted from sweet potato peel, white grape pomace and red grape pomace had a slightly higher dry weight of 0.58 g, 0.59 g and 0.61 g respectively. The dry weight of chitin extracted from molasses fermentation was much higher at 0.78 g. Chitin extracted from fermentation with potato peel had the highest dry weight of 0.89 g. The results suggest that the dry weight of extracted crude chitin samples may be directly influenced by the effectiveness of deproteinization and demineralization of prawn waste by fermentation.

**Table 1:** Dry weight of chitin extracted after fermentation on various carbon sources

Carbon source for fermentation	Dry weight (g)
Commercial chitin	N.A.
20 mL glucose in 80 mL water	0.50
20 mL molasses in 80 mL water	0.78
20 mL corn syrup in 80 mL water	0.38
15 g red grape pomace in 100 mL water	0.61
15 g white grape pomace in 100 mL water	0.59
15 g apple peel in 100 mL water	0.47
15 g pineapple peel and core in 100 mL water	0.57
15 g potato peel in 100 mL water	0.89
15 g mango peel in 100 mL water	0.55
15 g banana peel in 100 mL water	0.51
15 g sweet potato peel in 100 mL Water	0.58

### Fourier Transform Infrared Spectroscopy (FTIR) results

The DD% for commercial chitin from Sigma Aldrich and extracted crude chitin samples were determined using the formula as stated in section under Materials and Methods and summarized in Table 2. The calculations reflected that chitin extracted from banana peel fermentation had the highest DD% of 75.19%. Fermentation using molasses, red grape pomace and glucose fermentation displayed a high DD% of 73.98%, 72.90% and 72.57% respectively. DD% of chitin extracted from banana peel, molasses, red grape pomace and glucose fermentation exceeded that of commercial chitin (70.46%). Fermentation with corn syrup and white grape pomace had similar DD% of 65.21% and 65.92%. DD% of chitin extracted from potato peel of 54.75% and 53.51% from pineapple peel and core fermentation showed a much lower DD% than commercial chitin. Fermentation with apple peel had the lowest DD% of 53.39%. The results suggest that difference in the type of waste used as a carbon source during fermentation may affect the effectiveness of fermentation.

**Table 2:** Degree of deacetylation of chitin extracted after fermentation on various carbon sources

Carbon source for fermentation	Degree of deacetylation (%)
Commercial chitin	70.46
20 mL glucose in 80 mL Water	72.57
20 mL molasses in 80 mL water	73.98
20 mL corn syrup in 80 mL water	65.21
15 g red grape pomace in 100 mL water	72.90
15 g white grape pomace in 100 mL water	65.92
15 g apple peel in 100 mL water	53.39
15 g pineapple peel and core in 100 mL Water	53.51
15 g potato peel in 100 mL water	54.75
15 g mango peel in 100 mL water	60.50
15 g banana peel in 100 mL water	75.19
15 g sweet potato peel in 100 mL water	65.46

The FTIR spectra of commercial chitin exhibited a characteristic broad band at 3447 cm^−1^ attributing to O–H stretching. The absorption bands at 1660 cm^−1^ and 1559 cm^−1^ corresponded to Amide I C=O stretching and N–H bending and C-N stretching of Amide II (Barth 2007). The peak at 1073 cm^−1^ was due to the C–O–C vibration inside the chitin ring structure (Rumengan et al. 2014).

**Fig. 1:**
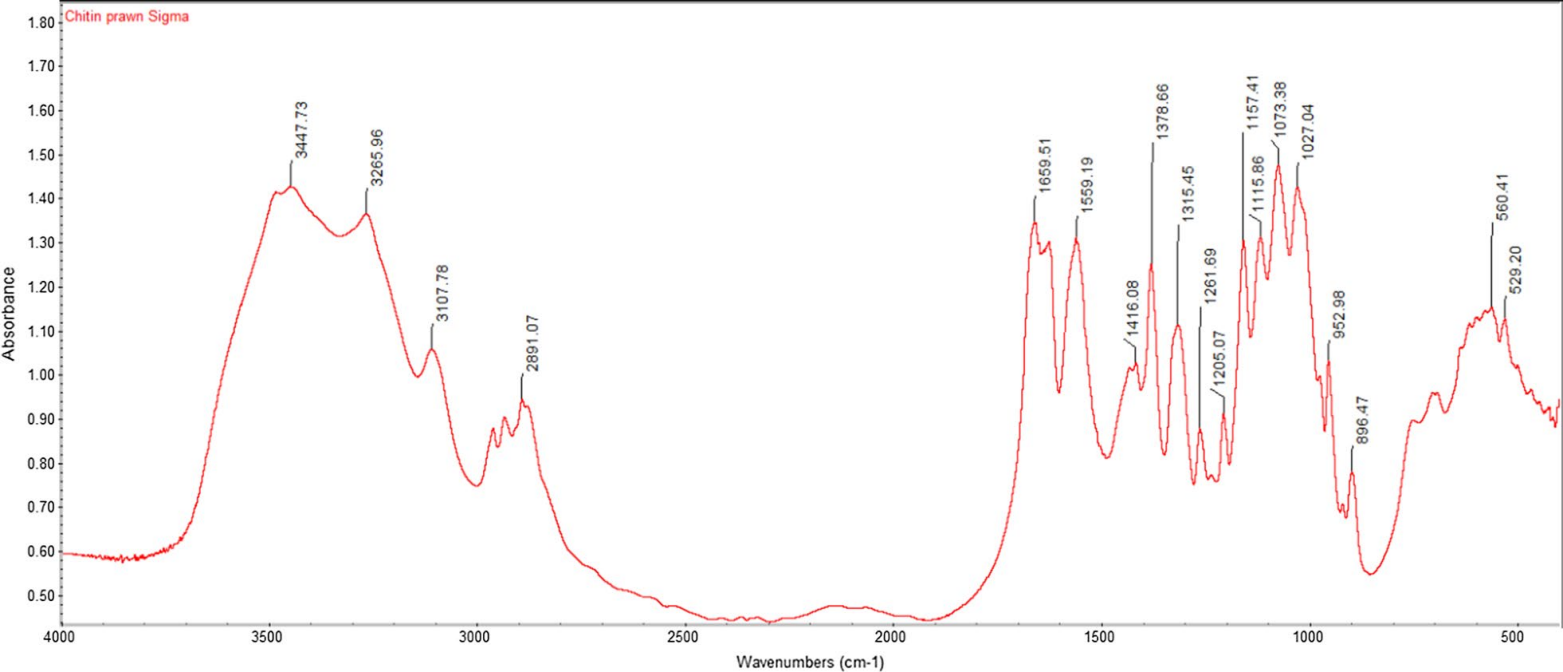
FTIR spectra of commercial chitin

**Fig. 2:**
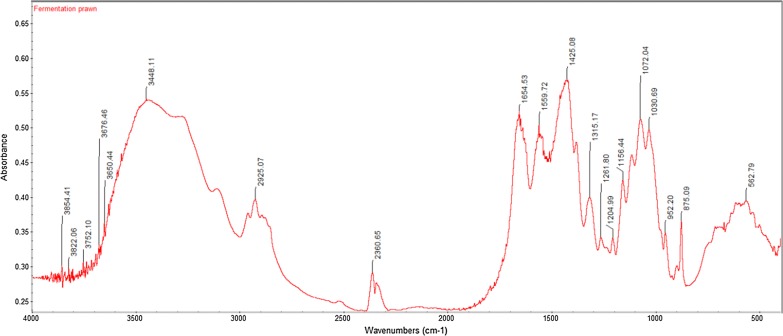
FTIR spectra of extracted chitin from glucose fermentation

**Fig. 3:**
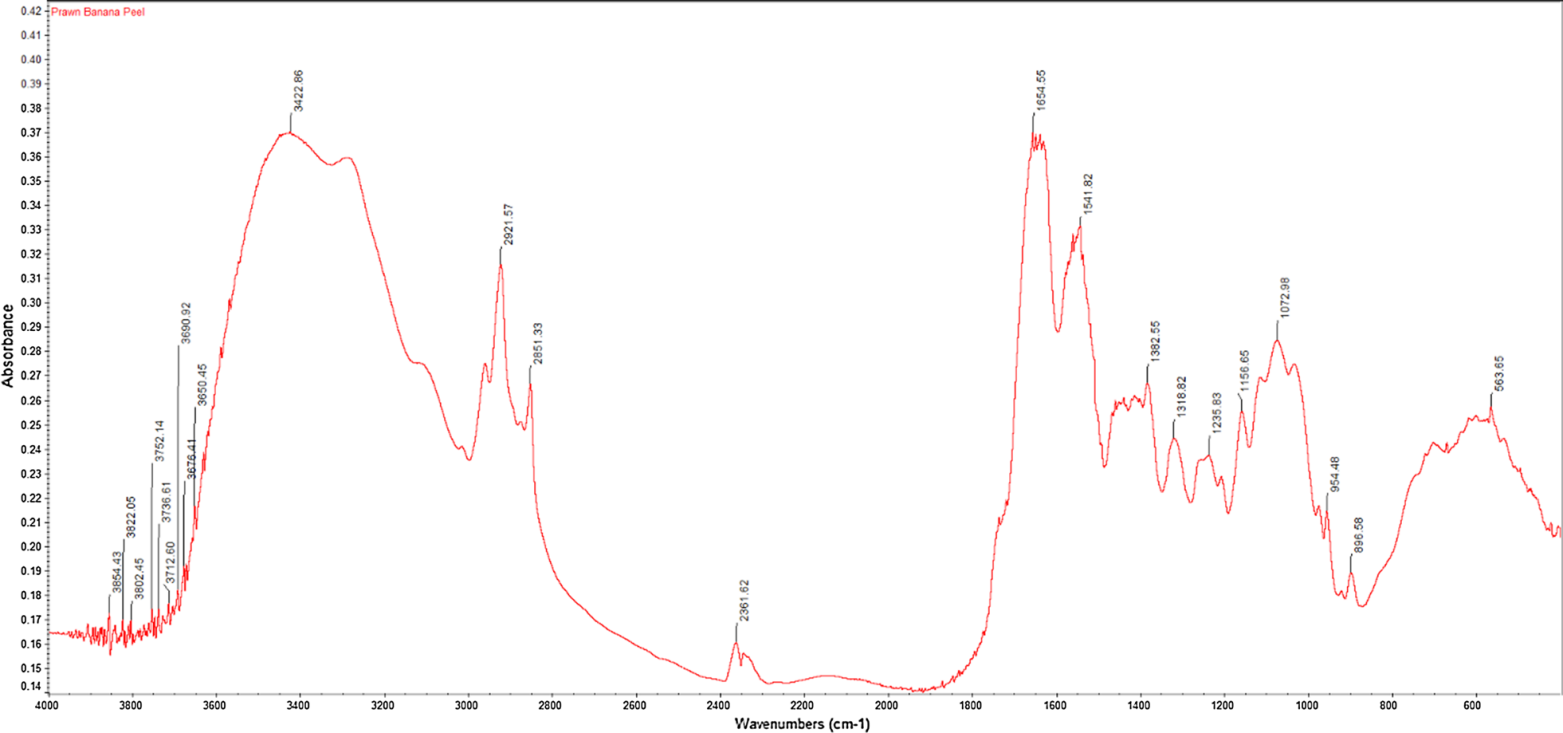
FTIR spectra of extracted chitin from banana peel fermentation

The FTIR spectra results from all extracted crude chitin samples displayed similar peaks to that of commercial chitin. The FTIR spectra for commercial chitin, chitin extracted from glucose and chitin extracted from banana peel fermentation with the highest DD% are shown in Figs. 1, 2, 3. Their respective adsorption bands are as indicated in Table 3.

**Table 3:** FTIR bands (cm^−1^) for commercial chitin, extracted chitin from glucose fermentation and from banana peel fermentation

Functional group	Commercial chitin	Extracted chitin from glucose fermentation	Extracted chitin from banana peel fermentation
O–H stretch	3447	3448	3423
C–H stretch	2891	2925	2921
C=O stretch of N-acetyl group (Amide I)	1660	1654	1655
N–H bend, C–N stretch (Amide II)	1559	1560	1542
CH_3_ in NHCOCH_3_ group	1379	1425	1383
CH_2_ wagging (Amide III)	1315	1315	1319
C–O–C stretch	1073	1072	1073

### Elemental analysis (EA) results

The results of the elemental analysis with the percentage of carbon, percentage of nitrogen and carbon/nitrogen ratio are shown in Table 4. The percentage of nitrogen was the highest for extracted chitin obtained from banana peel fermentation at 7.90%. Extracted chitin from sweet potato peel and molasses fermentation also had a high nitrogen at 7.71% and 7.29%. This was followed by extracted chitin from mango peel fermentation with percentage of nitrogen of 6.78%. These four carbon sources reflected a higher nitrogen content than commercial chitin (6.65%). Chitin extracted from corn syrup, red grape pomace and white grape pomace had a lower nitrogen content than commercial chitin at 6.17%, 6.08% and 6.00% respectively. Fermentation with glucose, pineapple peel and core and apple peel reflected a much lower percentage of nitrogen at 5.69%, 5.03% and 4.93% respectively. Chitin extracted from potato skin had the lowest percentage of nitrogen at 4.83%. Most of the crude chitin samples were observed to have a lower nitrogen content than the reference nitrogen content in fully acetylated chitin (6.89%) (de Alvarenga 2011).

**Table 4:** Elemental analysis of commercial chitin and chitin extracted after fermentation on various carbon sources

Carbon source for fermentation	Carbon (%)	Nitrogen (%)	Carbon/nitrogen ratio
Commercial chitin	46.12	6.65	6.94
20 mL glucose in 80 mL water	40.21	5.69	7.06
20 mL molasses in 80 mL water	45.50	7.29	6.24
20 mL corn syrup in 80 mL water	41.72	6.17	6.76
15 g red grape pomace in 100 mL water	41.63	6.08	6.85
15 g white grape pomace in 100 mL water	40.97	6.00	6.83
15 g apple peel in 100 mL water	41.62	4.93	8.44
15 g pineapple peel and core in 100 mL Water	34.27	5.03	6.81
15 g potato peel in 100 mL water	33.50	4.83	6.94
15 g mango peel in 100 mL water	46.89	6.78	6.92
15 g banana peel in 100 mL water	51.69	7.90	6.54
15 g sweet potato peel in 100 mL water	44.51	7.71	5.78

Commercial chitin and extracted chitin from potato peel fermentation had a similar carbon/nitrogen ratio of 6.94. Chitin extracted from apple peel and glucose fermentation showed a higher carbon/nitrogen ratio than commercial chitin of 8.44 and 7.06. The carbon/nitrogen ratio for extracted chitin from mango peel, red grape pomace and white grape pomace were comparable with that of commercial chitin (6.92, 6.85 and 6.83 respectively). Fermentation with pineapple peel and core and corn syrup had a slightly lower carbon/nitrogen ratio of 6.81 and 6.76. Chitin extracted from banana peel and molasses fermentation were measured to have a much lower carbon/nitrogen ratio of 6.54 and 6.24. The lowest carbon/nitrogen ratio was observed in extracted chitin from sweet potato peel fermentation of 5.78. The carbon/nitrogen ratios of all extracted crude chitin samples were similar to previous studies (Kumari et al. 2017).

### X-ray diffraction (XRD) results

**Fig. 4:**
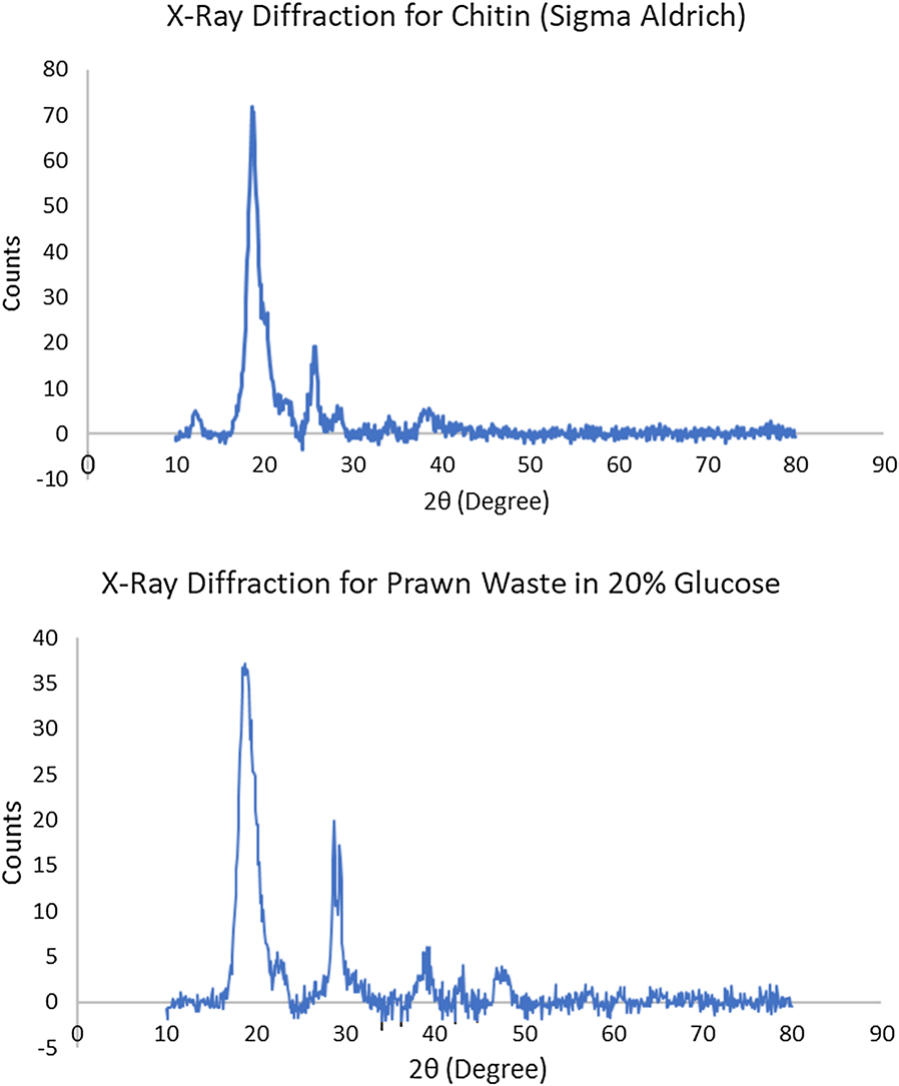
X-ray diffraction pattern of commercial chitin and extracted chitin from glucose fermentation

The extracted crude chitin samples showed a characteristic sharp peak at 20^o^ and two other weak peaks at 26° and 29° (Fig. 4), similar to the commercial shrimp chitin from Sigma Aldrich. The crystallinity index (CrI) of chitin calculated from the X-ray diffraction data showed that commercial chitin from Sigma Aldrich had a crystallinity of 87.56%. Chitin extracted from prawn waste fermented in 20% glucose solution surpassed the commercial chitin from Sigma Aldrich and showed the highest crystallinity at 98.16%. This was followed by fermentation in corn syrup at 97.99%, apple peel at 97.60%, mango peel at 97.34% and sweet potato peel at 96.08%. Chitin extracted from red grape pomace fermentation followed closely behind with a high crystallinity of 95.54%, while pineapple peel and banana peel fermentation also showed a high crystallinity of 94.28% and 93.51% respectively. Fermentation with molasses and white grape pomace had a slightly average crystallinity of 90.33% and 88.53% respectively. Only potato peel substrate registered a poor crystallinity of 79.89%. These results (Table 5) suggest that deacetylation and purification processes generally did not alter the natural crystallinity of prawn shell chitin and the high molecular weight of the extracted chitin may be responsible for the high crystallinity.

**Table 5:** degree of crystallinity of chitin extracted after fermentation on various carbon sources

Carbon source for fermentation	Degree of crystallinity (%)
Commercial chitin	87.56
20 mL glucose in 80 mL water	98.16
20 mL molasses in 80 mL water	90.33
20 mL corn syrup in 80 mL water	97.99
15 g red grape pomace in 100 mL water	95.54
15 g white grape pomace in 100 mL water	88.53
15 g apple peel in 100 mL water	97.60
15 g pineapple peel and core in 100 mL water	94.28
15 g potato peel in 100 mL water	79.89
15 g mango peel in 100 mL water	97.34
15 g banana peel in 100 mL water	93.51
15 g sweet potato peel in 100 mL water	96.08

### Statistical methods

The FTIR, EA and XRD experimental results were subjected to statistical analysis to evaluate their mean values, variances and standard deviations. Most of the data was found to conform to a small variance and narrow standard deviation, attesting to the accuracy of the measurements (Tables 6, 7 and 8).

**Table 6:** Statistical analysis of degree of deacetylation of chitin extracted after fermentation on various carbon sources

Carbon source for fermentation	Degree of de-acetylation (%)	Repeat 2	Mean	Variance	Standard deviation
Commercial chitin	70.46	70.77	70.62	0.05	0.22
20 mL glucose in 80 mL water	72.57	72.18	72.38	0.08	0.28
20 mL molasses in 80 mL water	73.98	72.65	73.32	0.88	0.94
20 mL corn syrup in 80 mL water	65.21	65.59	65.40	0.07	0.27
15 g red grape waste in 100 mL water	72.90	72.65	72.78	0.03	0.17
15 g white grape waste in 100 mL water	65.92	65.64	65.78	0.04	0.20
15 g apple pomace in 100 mL water	53.39	54.68	54.04	0.83	0.91
15 g pineapple peel and core in 100 mL water	53.51	54.14	53.82	0.20	0.44
15 g POTATO PEEL in 100 mL water	54.75	53.98	54.37	0.30	0.54
15 g mango peel in 100 mL water	60.50	61.00	60.75	0.13	0.35
15 g banana peel in 100 mL water	75.19	73.28	74.23	1.83	1.35
15 g sweet potato peel in 100 mL water	65.46	65.03	65.24	0.09	0.31

**Table 7:** Statistical analysis of elemental analysis of commercial chitin and chitin extracted after fermentation on various carbon sources

Carbon source for fermentation	C/N ratio	Repeat 2	Repeat 3	Mean	Variance	Standard deviation
Commercial chitin	6.94	6.91	6.90	6.91	0.00	0.02
20 mL glucose in 80 mL water	7.06	7.06	7.00	7.04	0.00	0.04
20 mL molasses in 80 mL water	6.24	5.95	6.35	6.19	0.05	0.22
20 mL corn syrup in 80 mL water	6.76	7.04	6.88	6.89	0.02	0.14
15 g red grape waste in 100 mL water	6.85	6.75	6.84	6.81	0.00	0.05
15 g white grape waste in 100 mL Water	6.83	6.85	6.77	6.82	0.00	0.04
15 g apple pomace in 100 mL water	8.44	6.99	7.11	7.51	0.65	0.80
15 g pineapple peel and core in 100 mL water	6.81	6.96	7.09	6.95	0.02	0.14
15 g potato peel in 100 mL water	6.94	7.10	6.99	7.01	0.01	0.08
15 g Mango Peel in 100 mL Water	6.92	6.74	6.53	6.73	0.04	0.19
15 g banana peel in 100 mL water	6.54	6.57	6.55	6.55	0.00	0.01
15 g sweet potato peel in 100 mL water	5.78	5.67	6.00	5.82	0.03	0.16

**Table 8:** Statistical analysis of degree of crystallinity of chitin extracted after fermentation on various carbon sources

Carbon source for fermentation	Degree of crystallinity (%)	Repeat 2	Mean	Variance	Standard deviation
Commercial chitin	87.56	86.86	87.21	0.25	0.49
20 mL Glucose in 80 mL water	98.16	97.53	97.85	0.20	0.45
20 mL molasses in 80 mL water	90.33	90.71	90.52	0.07	0.27
20 mL corn syrup in 80 mL water	97.99	96.73	97.36	0.79	0.89
15 g red grape waste in 100 mL water	95.54	94.64	95.09	0.41	0.64
15 g white grape waste in 100 mL water	88.53	89.69	89.11	0.67	0.82
15 g apple pomace in 100 mL water	97.6	96.48	97.04	0.63	0.79
15 g pineapple peel and core in 100 mL water	94.28	94.07	94.18	0.02	0.15
15 g potato peel in 100 mL water	79.89	82.11	81.00	2.46	1.57
15 g mango peel in 100 mL water	97.34	95.57	96.46	1.57	1.25
15 g banana peel in 100 mL water	93.51	92.86	93.19	0.21	0.46
15 g sweet potato peel in 100 mL water	96.08	95.29	95.69	0.31	0.56

## Discussion

Based on the experimental results obtained, the qualifying criteria for successful extraction of crude chitin from prawn waste at maximum preservation of its native structure was set at a degree of deacetylation above 70%, a carbon/nitrogen ratio above 6.80 and a degree of crystallinity above 90%. These criteria were met by commercial chitin from Sigma Aldrich, as well as when prawn waste was fermented with 20% glucose solution and red grape pomace. Red grape pomace performed excellently with a composition of soluble carbohydrates (mainly glucose and fructose) (Matthews et al. 1987). These soluble carbohydrates were easily broken down and fermentable to become simple sugars for use by lactic acid bacteria, with little waste residue left over. The remaining agro-industrial wastes consisted of a mixture of carbohydrate polymers, namely cellulose, hemi-cellulose and pectin and the non-carbohydrate polymer lignin (Production of Bioethanol from Fruit Wastes (Banana, Papaya, Pineapple and Mango Peels) Under Milder Conditions, 2018). Even though starch can be depolymerized to glucose units by enzymes (Sahni and Goel 2015), extraction of soluble sugars for fermentation from lignocellulosic biomass is more challenging due to the complex structure of lignocellulose (Doran-Peterson et al. 2008). Lactic acid bacteria ferment hexose sugars such as glucose by oxidizing NAD^+^ to NADH during glycolysis, thereby allowing pyruvate to serve as the electron acceptor to form lactate (Gänzle 2015). This study proves that though lactic acid bacteria are able to ferment a broad range of sugars from complex media, heterofermentative metabolism is not equally effective across all kinds of starch-based fermentations (Gänzle and Follador 2012). The sugar content in the food waste substrates used for our study contains natural sugars such as glucose, fructose and sucrose. Fermentation using carbon sources with high glucose content such as red grape pomace produced a higher quality of chitin. It is inferred from our study that glucose and fructose were utilized at different rates and to different extents by lactic acid bacteria during fermentation. Based on previous studies, it was reported that mannose-phosphotransferase catalyzes the transport and phosphorylation of sugars preferentially for glucose (monosaccharide) over sucrose (disaccharide has to be broken down into monosaccharides before sugars can be utilized), and the presence of fructose hindered the pathway for usage of glucose (Lu et al. 2001).

The dry weight of chitin is another indicator for the effectiveness of the removal of inorganic materials by demineralization from crustacean shells (Greene et al. 2016). Out of 5 g of prawn waste, 0.5–0.9 g of chitin was extracted across all fermentations, translating to a yield of 10–20% comparable with previous studies (Bahasan et al. 2017). From the EA results, it can be seen that the nitrogen content of chitin extracted from prawn waste fermentation across most of carbon sources were much lower than fully acetylated chitin (6.89%). This implies efficient deproteinization as a lower nitrogen content indicates minimal residual protein in the crude extracted chitin (Ibitoye et al. 2018). In our study, the carbon/nitrogen ratios for most of the extracted crude chitin were lower than commercial chitin (6.94). A lower carbon/nitrogen ratio suggests effective demineralization of prawn waste. According to previous studies, the presence of calcium carbonate in composting substrate speeds up the rate of decomposition (Rusmini and Daryono 2017). With high levels of calcium carbonate in prawn shells, rate of decomposition by fermentation would be high (Arbia et al. 2017).

In addition, the DD% determined from the FTIR results also signifies successful chitosan extraction. With all crude extracted chitin from different fermentation carbon sources having a degree of deacetylation higher than 50% or a degree of acetylation lower than 50%, it is concluded that all obtained crude extracted chitin samples contain chitosan. Chitosan obtained from the partial deacetylation of chitin becomes soluble in aqueous acidic medium once the degree of acetylation falls below 50% (Roy et al. 2017). Due to poor solubility of chitin, chitin has more biological applications when partially deacetylated to chitosan and is more easily processed into various biomaterials (Ibrahim and El-Zairy 2015). Therefore, red grape pomace with a high DD% (72.90%) is selected as an excellent waste substrate to produce highly purified chitosan. Although banana peel achieved a high DD% (75.90%), the residual nitrogen content was 7.90%, which is higher than the N% of fully acetylated chitin, implying a less effective deproteinization. However it is remarkable to note that banana peel has the lowest cellulose content (8.6%) (Yusuf et al. 2019) out of all the lignocellulosic waste substrates (average 20–30%) (Jahid et al. 2018; Khan 2017). The banana peel contains small amounts of cellulose, hence enzymatic hydrolysis is made more readily accessible due to the high content of pectins and sugars, with lower hemicellulose and lignin content (Szymanska-Chargot et al. 2017).

Lastly, from the values of the degree of crystallinity of determined from the XRD results, it can be seen that extracted crude chitin from fermentation across all carbon sources had a high crystallinity above 80%. This proves that the extracted crude chitin retained its orderly crystalline microfibril structure similar to its native state in the exoskeleton of arthropods (Pacheco et al. 2011).

Factors that affect the fermentation process and deproteinization and demineralization efficiencies include—shrimp shell concentration; type of carbon source and concentration; species and quantity of inoculums; duration of fermentation; initial medium pH; incubation temperature; speed of agitation and volume of culture (Rao et al. 2000). However, this study mainly explores the effect of carbon source to optimize deproteinization and demineralization efficiencies in prawn shells. Future work could possibly include the use of response surface methodology or Plackett–Burman factorial design to optimize other fermentation parameters in order to further decrease any residual protein or mineral impurity levels in crude extracted chitin.

## Data Availability

Data and materials would be available on request.
